# Sexual Dysfunction in Female Patients Undergoing Hemodialysis and Its Relationship With Anxiety and Depression

**DOI:** 10.7759/cureus.29883

**Published:** 2022-10-03

**Authors:** Syed Asfand Yar Shah, Waseem Sajjad, Wajih Ul Hassan, Usama Bin Shabbir

**Affiliations:** 1 Internal Medicine, Nishtar Medical University, Multan, PAK

**Keywords:** multan, depression, anxiety, sexual dysfunction, hemodialysis

## Abstract

Introduction

Hemodialysis is renal replacement therapy. However, it is associated with various complications. Sexual dysfunction is one of them. It is estimated that 25%-64% of female patients on hemodialysis have sexual dysfunction worldwide. This impaired quality of life further leads to anxiety and depression. Around 22.8%-39.3% of patients on hemodialysis are depressed while 27% have a major anxiety disorder. In Pakistan, the incidence of end-stage renal disease (ESRD) is 100 per million people. As far as we know, this is the first study conducted in Multan to assess sexual dysfunction in female patients undergoing hemodialysis. The objective of this study was to find out the prevalence of sexual dysfunction in female patients on hemodialysis and its relationship with anxiety and depression.

Material and methods

A cross-sectional study was conducted in Nishtar Medical University Hospital, Multan, Pakistan, from February 2021 to May 2021. Data were collected from 55 female patients in the form of an interview after having met inclusion and exclusion criteria. The questionnaire had some sociodemographic questions. The female sexual function index (FSFI) was used to evaluate sexual dysfunction in hemodialysis patients while anxiety and depression were assessed using the hospital anxiety and depression (HADS) scale. SPSS version 25.0 (IBM Corp., Armonk, NY) was used for data analysis. Pearson's correlation was used to find the association of sexual dysfunction with anxiety and depression. A p-value of <0.05 was considered statistically significant.

Results

Hypertension (56.4%) was the leading cause of chronic kidney disease (CKD). The most serious problem of the patients was sexual arousal suggested by the low mean score (1.77 ± 2.27) as compared to other sexual domains. Anxiety and depression were found in 20% and 30.9% of patients, respectively. There was a significant negative correlation between age ((arousal: r= -0.297, p= 0.028), (lubrication: r= -0.274, p= 0.043), (orgasm: r= -0.298, p= 0.027), and (pain: r= -0.271, p= 0.045)) and depression ((desire: r= -0.465, p= <0.001), (satisfaction: r= -0.366, p= 0.006)) with sexual function. While anxiety was not significantly associated with sexual function ((desire: r= -0.347, p= 0.069), (arousal: r= 0.053, p= 0.700), (lubrication: r= 0.061, p= 0.658), (orgasm: r= 0.047, p= 0.736), (satisfaction: r= -0.113, p= 0.410) and (pain: r= 0.045, p= 0.746)).

Conclusion

Sexual dysfunction is not uncommon in female hemodialysis patients. There was a significant negative correlation of different sexual domains with age and depression, respectively. The correlation of anxiety with sexual dysfunction was found to be statistically insignificant.

## Introduction

Hemodialysis is a form of renal replacement therapy for end-stage renal failure patients. Although life-saving, it is associated with multiple complications such as dialysis disequilibrium syndrome, hemolysis, allergic reactions, and intradialytic hypotension [1]. Furthermore, hemodialysis significantly impairs the quality of life, resulting in a high symptoms burden [2,3]. Sexual dysfunction is another important negative outcome of end-stage renal disease patients receiving dialysis. Sexual dysfunction in men receiving hemodialysis is well-recognized in the literature. On average, 20% to 87.7% of the men with end-stage renal disease on hemodialysis have been reported to have erectile dysfunction [4]. Compared to men, sexual dysfunction in female hemodialysis patients is less well understood and researched [5]. The incidence of sexual dysfunction in female hemodialysis patients worldwide is approximately 25%-64% [6]. Lack of libido, dyspareunia, vaginal dryness, minimal sense of arousal, and anorgasmia are common symptoms of female sexual dysfunction [7].

Regular dialysis is extremely distressing. Besides impairing quality of life, it could lead to depression and anxiety among dialysis patients. Among patients with end-stage renal disease (ESRD), depression is the most common psychiatric illness. The reported prevalence of depression in the dialysis population varied from 22.8% (interview-based diagnosis) to 39.3% (self- or clinician-administered rating scales) [8]. Another study conducted in urban hemodialysis patients in the US in 2007 in predominantly black patients using Beck's Depression Inventory (BDI) scale showed that 29% had a current depressive disorder: major depression was found among 20% of the patients, and 9% had a diagnosis of dysthymia or depression not otherwise specified [9].

Depression is characterized by a persistent depressed (low) mood, feelings of worthlessness, a lack of positive affect, and a loss of interest in things one once enjoyed (anhedonia). It is a significant prognostic factor for mortality among dialysis patients (associated with a 50% increase in risk) [10]. In addition, it is associated with an increased risk of hospitalization, treatment nonadherence (shortened and skipped treatments), inflammation, and malnutrition in patients receiving dialysis [10].

In Pakistan, the estimated incidence of ESRD is 100 per million of the population [11]. However, despite the increasing number of ESRD patients who require dialysis in Multan, little or no studies have been conducted to evaluate sexual dysfunction in female patients undergoing dialysis and understand how depression and anxiety affect the sexual health of such patients. Therefore, the objective of this study is to identify the prevalence of sexual dysfunction in female hemodialysis patients and its connection with depression and anxiety.

## Materials and methods

A cross-sectional study was carried out in the dialysis unit of the Nephrology Department, Nishtar Medical University Hospital, Multan, Pakistan, from February 2021 to May 2021. Ethical approval was given by the ethical review committee of Nishtar Hospital Multan. Data were collected after obtaining proper informed consent, by distributing the questionnaire among study participants using the random sampling technique. We divided the questionnaire into three sections. Section A comprised eight questions regarding various sociodemographic characteristics (age, BMI, education, and occupation) and a few clinical questions (cause of renal failure, associated comorbidity, duration of hemodialysis, and hemoglobin).

We included only those female patients aged 19 to 60 years who were married and living with their husbands for the past four weeks. In contrast, the study did not include female patients who were unmarried, widows, living away from their husbands, or suffering from some major psychiatric illnesses.

There were 19 questions in section B related to sexual functioning in female patients-FSFI [12]. Six sexual domains that include desire, arousal, lubrication, orgasm, pain, and satisfaction, each with a maximum score of 6, were assessed over the period of four weeks to construe the presence of sexual dysfunction if scores were equal to or below 26.55 [12].

Section C was related to questions that assess the severity of anxiety and depression among participants using the Hospital Anxiety and Depression Scale (HADS) [13]. It has seven questions on depression and anxiety, respectively. A score of 0 to 7 for each characteristic was regarded as normal; 8 to 10 as borderline; and 11 to 21 as abnormal.

SPSS version 25.0 (IBM Corp., Armonk, NY) was used to analyze the data. Frequencies, means, and standard deviations were calculated for various sociodemographic and clinical variables. Pearson's correlation was used to find the relationship between various categorical variables. A p-value of <0.05 was considered statistically significant.

## Results

In the study, 55 female patients receiving hemodialysis were included. Among these patients, the minimum age was 19 years, and the maximum age was 60 years. The mean duration of hemodialysis was 31.52 ± 30.2 months, and their average BMI was 22.01 ± 5.28. Among these, 22 (40%) were illiterates, 18 (32.7%) got education up to primary level, 9 (16.4%) up to secondary level, and 6 (10.9%) went to university. While 53 (96.4%) were housewives. The leading cause of renal failure was hypertension (56.4%) followed by diabetes mellitus (12.7%) and postpartum hemorrhage (12.7%). About 30 (54.5%) patients had associated hepatitis C and two (3.6%) had associated cardiovascular disease. The average hemoglobin was 9.25 ± 1.85 (Table 1).

**Table 1:** Demographic and clinical parameters of patients (n=55)

VARIABLES	n (%)
Patient's demographics	
Age	19-60
BMI (kg/m2) ± SD	22.01 ± 5.28
Education	
Illiterate	22 (40%)
Primary	18 (32.7%)
Secondary	9 (16.4%)
University	6 (10.9%)
Occupation	
Working	2 (3.6%)
Housewives	53 (96.4%)
Causes of Renal Failure	
Diabetes Mellites	7 (12.7%)
Hypertension	31 (56.4%)
Glomerulonephritis	1 (1.8%)
Cystic Kidney Disease	2 (3.6%)
Renal Stone	1 (1.8%)
Post-Partum Hemorrhage	7 (12.7%)
Unknown Causes	6 (10.9%)
Associated Diseases	
Hepatitis C	30 (54.5%)
Cardiovascular Diseases	2 (3.6%)
No Associated Disease	23 (41.8%)
Duration of Hemodialysis in Months ± SD	31.52 ± 30.2
Hemoglobin (g/l)	9.25 ± 1.85

Considering the domains of sexual activity, the mean ± SD, median, and total score are listed in Table 2.

**Table 2:** Score distribution of each domain of sexual function

Domain	Mean ± SD	Median	IQR
Desire	2.38 ± 1.31	2.4	2.4
Arousal	1.77 ± 2.27	0.00	3.9
Lubrication	2.43 ± 2.83	0.00	6.0
Orgasm	2.09 ± 2.52	0.00	5.2
Satisfaction	2.64 ± 1.87	2.0	4.0
Pain	2.43 ± 2.87	0.00	6.0
Total Score	13.70 ± 12.83	4.6	26.3

**Figure 1:**
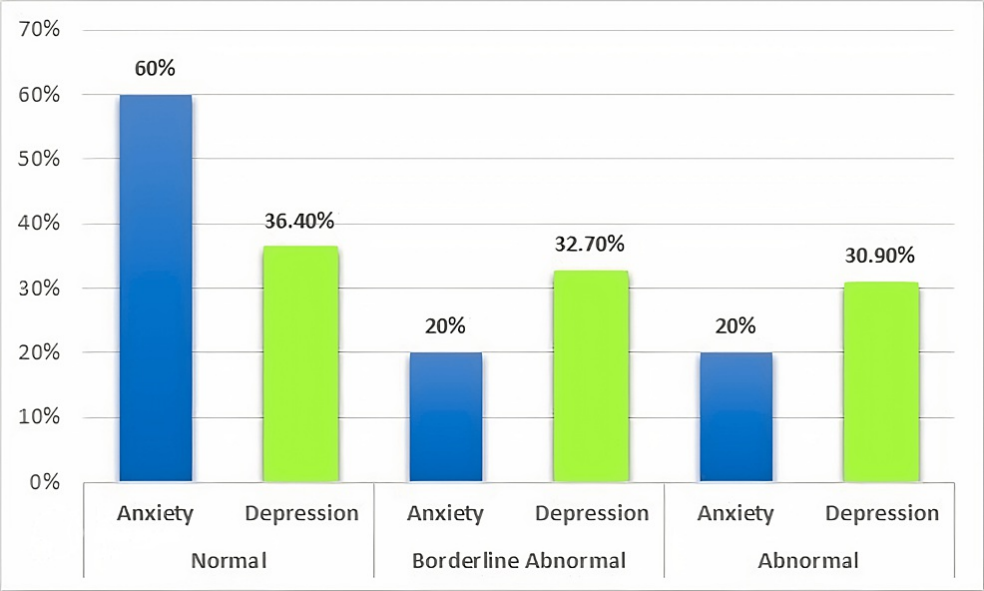
Severity of anxiety and depression in patients undergoing hemodialysis

Patients were assessed for anxiety and depression using the HADS score ranging from 0 to 21. Of all 55 patients accessed for anxiety, 33 (60%) were normal, 11 (20%) were borderline abnormal, and 11 (20%) were abnormal. Concerning depression, 20 (36.4%) were normal, 18 (32.7%) were borderline abnormal, and 17 (30.9%) were abnormal (Figure 1).

The final result shows a significant negative correlation of age with arousal, lubrication, orgasm, and pain. There is also a significant negative correlation between desire and satisfaction domain with depression abnormal. However, no sexual domain is correlated significantly with borderline depression and anxiety (Table 3).

**Table 3:** Pearson's correlation coefficient results of age, depression, and anxiety with sexual function domains * co-relation is significant at 0.05 level (2 tailed)

	Desire Score	Arousal Score	Lubrication Score	Orgasm Score	Satisfaction Score	Pain Score
Age	r= -0.257	r= -0.297*	r= -0.274*	r= -0.298*	r= -0.206	r= -0.271*
	p= 0.058	p= 0.028	p= 0.043	p= 0.027	p= 0.132	p= 0.045
Depression						
Normal	r= -0.286	r= -0.078	r= -0.018	r= -0.086	r= -0.309	r= -0.038
	p= 0.34	p= 0.573	p= 0.896	p= 0.531	p= 0.22	p= 0.782
Borderline Abnormal	r= 0.165	r= 0.058	r= 0.115	r= 0.067	r= 0.043	r= 0.122
	p= 0.230	p= 0.673	p= 0.405	P= 0.629	p= 0.753	p= 0.376
Abnormal	r= -0.465*	r= -0.140	r= -0.135	r= -0.157	r= -0.366*	r= -0.166
	p= <0.001	p= 0.308	p= 0.326	p= 0.251	p= 0.006	p= 0.234
Anxiety						
Normal	r= 0.180	r= -0.156	r= -0.147	r= -0.171	r= 0.002	r= -0.156
	p= 0.188	p= 0.255	p= 0.283	p= 0.212	p= 0.991	p= 0.254
Borderline Abnormal	r= 0.026	r= 0.138	r= 0.119	r= 0.163	r= 0.111	r= 0.147
	p= 0.851	p= 0.316	p= 0.386	p= 0.235	p= 0.418	p= 0.285
Abnormal	r= -0.347	r= 0.053	r= 0.061	r= 0.047	r= -0.113	r= 0.045
	p= 0.069	p= 0.700	p= 0.658	p= 0.736	p= 0.410	p= 0.746

## Discussion

Sexuality is one of the most complex areas of human behavior. Besides its emotional aspect, it has sociological, behavioral, and biological aspects. Sexual activity has a positive role in relaxation, mood elevation, and boosting our immune system [14]. Sexual dysfunction is the inability to react emotionally or physically to sexual stimulation. Multiple factors, physical or psychological, affect sexual functioning. Any chronic illness leads to body fatigue, malaise, hormone changes, and psychological or behavioral alternations resulting in sexual dysfunction. Any mental or physical condition that affects the quality of life also can affect sexual desire and function. In the current study, 55 female patients on hemodialysis were assessed for their sexual functioning in the last four weeks using a 19-item self-administered questionnaire-FSFI [12].

In our study, hypertension was the most common comorbidity (56.4%) in chronic kidney disease (CKD) patients. Hypertension was also the most common risk factor as found by Strippoli et al. (67%) [5], Gatmiri et al. (40.5%) [6], Yaqoob et al. (81.3%) [7], Saglimbene et al. (60%) [15], and Zeighami et al. (61.2%) [16]. This is because hypertension is the most commonly prevalent comorbidity in the general population (30%) [17].

We found that the most serious problem of the patients was sexual arousal suggested by the low mean score (1.77 ± 2.27) as compared to other sexual domains while, at the same time, most were sexually satisfied, suggesting that sexual satisfaction does not rely merely upon sexual desire and arousal. It is multidimensional and affected by various physical, psychological, and social factors. This finding is supported by other studies as well [7,16]. Moreover, some authors described pain as the least common problem [18,19].

Many studies reported a negative correlation between age and sexual function with a slight difference in various domains [5-7]. We also found a significant negative correlation between age and all the sexual domains except desire and satisfaction. It means that as the patient gets older, arousal, lubrication, orgasm, and pain decrease. This could be because of physiological hormonal changes affecting sexual functioning.

A chronic illness not only affects the body physically but also affects its psychological, social, and financial aspects, further deteriorating one's mental health. Using HADS, 20% of patients on hemodialysis had anxiety, and 30% of patients were found depressed in this study. A similar study conducted in Karachi reported a slightly higher percentage of anxiety (45.8%) and depression (31.3%) [7]. In Iran, 48.6% had anxiety/depression [6]. As a result, anxiety/depression further ruins one's sexual life. In the current study, depression abnormally has a significant negative correlation with desire and satisfaction, meaning that as the patient becomes depressed, his libido and sexual satisfaction are significantly reduced. We found no significant correlation between anxiety and sexual domains, a finding that is consistent with other studies [5,15,18]. Mohamed BW and his colleagues found no correlation between depression/anxiety with sexual domains [6]. On the other hand, Yaqoob et al. found a significant negative correlation between borderline depression and all sexual domains (except desire) [7]. This could simply be because of regional variations in the social support system and duration of illness.

There were, however, certain limitations to our study. First, we had a small sample size. Second, it was a single-center study. Third, a pre-dialysis psychological evaluation was not done. Fourth, many other risk factors of sexual dysfunction were not excluded.

## Conclusions

Sexual dysfunction was a common problem in female hemodialysis patients. Most of them had problems with arousal. There was a significant negative correlation between different sexual domains with age and depression. The correlation of anxiety with sexual dysfunction was found to be statistically insignificant. Our study underscores the importance of psychological interventions besides pharmacological therapy in hemodialysis patients.
